# Barotropic modes, baroclinic modes and equivalent depths in the atmosphere

**DOI:** 10.1002/qj.3781

**Published:** 2020-04-20

**Authors:** Yair De‐Leon, Nathan Paldor, Chaim I. Garfinkel

**Affiliations:** ^1^ Ring Department of Atmospheric Sciences, Fredy and Nadine Herrmann Institute of Earth Sciences The Hebrew University of Jerusalem Jerusalem Israel

**Keywords:** baroclinic modes: definition, barotropic modes: definition, equivalent depth, vertical structure function

## Abstract

The equivalent depth of a rotating fluid layer determines the phase speed of all free (i.e. unforced) linear waves that propagate in it. The equivalent depth can be estimated from the eigenvalues of a second‐order differential equation for the vertical dynamics where the mean temperature profile is a coefficient. The eigenfunctions dictate the vertical structure of the modes. This work combines analytic and numerical solutions of the vertical structure equation for various mean temperature profiles and for various combinations of top and bottom boundary conditions relevant to two atmospheric configurations: troposphere and combined troposphere‐stratosphere. Our formulation provides a clear definition of the barotropic mode and the countable baroclinic modes (including the special *n* = 0 mode). The barotropic mode exists only when the lower boundary condition is the vanishing of the vertical velocity (i.e. when a solid boundary bounds the layer from below) and the equivalent depth of this mode is about 10 km. The *n* = 0 baroclinic mode does not exist in a layer whose thickness exceeds a threshold value of about 20 km and therefore this mode does not exist in the combined troposphere‐stratosphere layer. The upper boundary condition affects the eigenvalues much more so than the details of the temperature profile, as the details of the temperature profile only affect the equivalent depth in the troposphere. Increasing the height of the upper boundary has little effect on the barotropic mode, but strongly influences the phase speed and vertical structure of the baroclinic modes; a general circulation model with a lid at or below the stratopause where, for example, planetary Rossby waves are present will therefore be incapable of correctly simulating the interaction of these waves with the mean flow. The values of the equivalent depth of baroclinic modes are approximately 10% of the actual layer's depth in all realistic cases.

## Graphical abstract

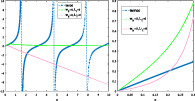

## 1 INTRODUCTION

Wave solutions of the linearized primitive equations form the basis of our understanding of atmospheric dynamics, as they provide a simple description of how variability in one region of the atmosphere can affect other regions. The primitive equations can be recast as two distinct, but coupled, sets of equations if the zonal wind is either constant or a function of latitude only (Winter and Schmitz, 1998): the shallow‐water equations (SWE) for the two‐dimensional (2D) horizontal dynamics, and an eigenvalue equation for the 1D vertical structure problem. These two sets are coupled via a separation constant denoted by *h*_*n*_ and known as the equivalent depth (where the subscript *n* is the vertical mode number). For certain applications of the primitive equations (e.g. tides), the frequency, *ω*, of the horizontal waves is assumed to be known due to presence of forcing (e.g. tidal forcing: Lindzen and Chapman, 1969) so by applying appropriate horizontal boundary conditions the SWE can be solved to yield *h*_*n*_ which is then substituted in the vertical structure equation. The resulting parameter‐free equation is solved to yield the vertical structure function (which is proportional to the vertical velocity in pressure coordinates, denoted in Lindzen and Chapman (1969) by *L* and this notation is adopted in the present work as well) by specifying boundary conditions at the layer's bottom and top boundaries. In this approach the vertical boundary conditions only affect the vertical structure function but not the equivalent depth, *h*_*n*_.

When no forcing affects the dynamics in the problem, that is, for free waves, the 2D SWE can determine either the separation constant, *h*_*n*_, or the frequency, *ω* (related to the phase speed), while the second parameter is treated as a free parameter. This approach was employed in De‐Leon and Paldor (2011) and Paldor *et al*. (2013) to calculate the waves' phase speeds (as well as the meridional structure of their amplitudes) as a function of the free parameter *h*_*n*_. Since the 2D horizontal equation in the unforced case determines only the functional relation between *h*_*n*_ and the frequency, *ω* (or the phase speed), the value of *h*_*n*_ has to be determined from solutions of the 1D vertical equation in which *h*_*n*_ appears as the eigenvalue of a second‐order eigenvalue problem. Solutions of the vertical structure equation can be derived only by specifying a mean temperature profile and the associated boundary conditions. This 1D eigenvalue problem for the vertical structure can be solved analytically when the temperature profile is sufficiently simple (cf. Section 4.2 of Andrews *et al*., 1987) and numerical solutions can be calculated for more complex temperature profiles. Once the vertical structure equation is solved and the set of possible eigenvalues (i.e. the equivalent depths, *h*_*n*_) computed, the horizontal SWE can be solved to yield the frequency, *ω*. As the properties of the horizontal wave modes depend sensitively on the equivalent depth (see De‐Leon and Paldor (2011) for small *h*_*n*_ and Paldor *et al*. (2013) for large *h*_*n*_), it is crucial to understand the factors that govern the equivalent depth.

Specifically, we wish to quantify and estimate the dependence of eigenvalues of the vertical structure equation on the mean temperature profile and on the boundary conditions that must be applied in order to solve the differential equation. While the natural and physical lower boundary condition at the bottom of the layer (which is the solid ground) is *w* = 0 (where *w* = *dz*/*dt* is the vertical velocity), for the upper boundary condition, *w* = 0 is realistic only at infinity (i.e. in outer space). For sufficiently simple temperature profiles, the vertical structure equation can be solved analytically by imposing a radiation condition at the top of the layer in which only upward propagating wave solutions are selected. If the temperature profile is more complex, then numerical solutions are needed to solve the vertical structure equation. However, numerical solutions cannot readily incorporate such a radiation boundary condition, since this condition only eliminates one of two solutions of a second‐order differential equation after the two independent solutions of the equation have been found (e.g. a solution in which the energy is unbounded).

Instead, a wide variety of approaches have been used historically to set boundary conditions in numerical solutions. For example, Chen and Robinson (1992) use a model with a log‐*p* vertical coordinate and the boundary condition [*d*/*dz* + 1/(2*H*)]Φ = 0 (where Φ is the geopotential and *H* is the scale height) is applied at the upper lid located at 60 km. Nigam and Lindzen (1989) use geometric height as their vertical coordinate, and impose χ(49 km) = χ(50 km) where χ = *p*/[*g* ⋅ (basic state density)] with their model lid at 50 km. Jacqmin and Lindzen (1985) impose a similar upper boundary to Nigam and Lindzen (1989). Lin (1982) uses a model with a log‐*p* vertical coordinate and applies Φ = 0 at his model lid. (Most of these studies also apply a sponge layer below their model top to avoid wave reflection that can arise from such an upper boundary.) It is clear that there is a wide variety of approaches to specifying the top boundary conditions.

There is a similar variety of approaches among modern general circulation models as to the height at which the upper boundary condition is applied. There is substantial evidence that modern general‐circulation models (GCMs) with a lid within the stratosphere or at the stratopause (i.e. low‐top) behave differently than those with a lid well above the stratopause (high‐top). Low‐top models, on average, simulate too few stratospheric sudden warmings as compared to high‐top models (Charlton‐Perez *et al*., 2013). The low‐top models are apparently incapable of fully resolving the wave dynamics that eventually weakens the vortex. Many of these low‐top models also simulate a systematically different response to climate change as compared to high‐top models (Scaife *et al*., 2012; Manzini *et al*., 2014). Thus, it is of importance to establish the effect of the upper boundary on wave modes.

We also wish to provide a clear classification of the different types of modes of the solutions of the vertical structure equation encountered in the literature: the barotropic mode, the *n* = 0 baroclinic mode (a.k.a. first baroclinic mode) and the *n* ≥ 1 baroclinic modes. Tropical waves forced by convective maxima in the mid‐troposphere will project strongly onto a vertical mode with a half‐wavelength of ∼14 km in the troposphere, or *h*_*n*_ = 200 m (Garcia and Salby, 1987; Kiladis *et al*., 2009) with low mode numbers (for example, *n* = 1 baroclinic structure), and hence a better understanding of the theoretical properties of waves with low mode numbers could help us interpret the tropical wave spectrum. Examination of the conditions for the existence of a barotropic mode and its characteristics (see details in Section 3) is of importance to the understanding of barotropic sudden stratospheric warming (SSW), and which some authors (e.g. Tung and Lindzen, 1979; Plumb, 1981; Esler and Scott, 2005; Matthewman and Esler, 2011) link to resonance of a pre‐existing wave. Some observed vortex split events extend down nearly into the troposphere (e.g. the SSWs in 1979 and 2009), but others do not (Matthewman *et al*., 2009). Is this resonance solely within the stratosphere or is it coupled to the troposphere?

In this article, we solve the vertical equation for two atmospheric configurations: troposphere and combined layer of troposphere‐stratosphere to yield the eigenfunction, *L*_*n*_, and the equivalent depth, *h*_*n*_ (and with it the square of the speed of gravity waves, *gh*_*n*_) in each layer. Since there is no natural upper boundary condition in a bounded layer of the atmosphere (other than the radiation condition) and in an unbounded atmosphere the primitive equations themselves are no longer valid (Cohn and Dee, 1989), we assess the sensitivity of the solutions to the Dirichlet, Neumann or Robin (i.e. a linear combination of Dirichlet and Neumann conditions) boundary conditions imposed at a given height, where we expect a discrete (i.e. discontinuous) spectrum of solutions. We also assess the sensitivity of the solutions to the height at which these boundary conditions are imposed. The case of the stratosphere layer that has no solid lower boundary is more involved and is analysed in Appendix B.

After first reviewing the governing equations and boundary conditions and introducing the temperature profiles used (Section 2), we show how the governing equation can be recast in such a way as to clearly indicate which modes are barotropic and which are baroclinic and discuss the conditions under which analytic and semi‐analytic solutions can be obtained (Section 3). We then introduce the methods of numerical solution (Section 4), and present results on the sensitivity of the eigenfunctions and eigenvalues (i.e. equivalent depths) to the temperature profile and boundary conditions (Section 5). We summarize our results and discuss their implications in Section 6.

## 2 GOVERNING EQUATIONS, TEMPERATURE PROFILES AND BOUNDARY CONDITIONS

### 2.1 The vertical structure equation

The primitive equations in spherical coordinates (and without zonal mean flow, see the discussion in Section 6 below) are traditionally separated into two sets of coupled (via the separation constant) 2nd‐order equations for the material derivative of the pressure: one for the vertical dynamics and one for the horizontal dynamics (e.g. Taylor, 1936; Lindzen and Chapman, 1969; Staniforth *et al*., 1985; Daley, 1991; Winter and Schmitz, 1998). Regardless of the coordinate system used in the horizontal equations, the vertical equation is:

(1) $$\frac{\mathrm{RT}}{g}\frac{d^{2}L_{n}}{dz^{2}}+\left(\frac{R}{g}\frac{\mathrm{dT}}{\mathrm{dz}}-1\right)\frac{dL_{n}}{\mathrm{dz}}+\frac{1}{h_{n}}\left(\frac{R}{g}\frac{\mathrm{dT}}{\mathrm{dz}}+\kappa \right)L_{n}=0,$$

where *R* is the gas constant, *g* is the gravitational acceleration, *z* is the vertical coordinate, *T* = *T*(*z*) is the vertical temperature profile (denoted in Lindzen and Chapman (1969) by *T*_0_(*z*)), $\kappa =\frac{\gamma -1}{\gamma }=\frac{2}{7}$ where *γ* is the ratio between the specific heat capacities in constant pressure and constant volume. *h*_*n*_ (where the subscript *n* is the vertical mode number) is the value of the separation constant (between the horizontal and vertical parts) also known as the equivalent depth, which is determined by considering density changes via the temperature profile, so *gh*_*n*_ varies accordingly (while *g* remains unchanged). The unknown function *L*_*n*_(*z*) is the *n*th mode vertical structure function proportional to the vertical velocity in pressure coordinates (cf. eqs 16 and 27 in Ch. 3 of Lindzen and Chapman, 1969). Equation 1 can be obtained from eq. 27 in Ch. 3 of Lindzen and Chapman (1969) by setting *J* = 0 and *T*(*z*) = *gH*(*z*)/*R* and it is the vertical structure equation in the unforced problem, that is, in the absence of heating or gravitational tidal forcing (thus, there are no *s* or *σ* superscripts in *L*_*n*_). As shown in Winter and Schmitz (1998), the derivation is identical if zonal wind is present, as long as the zonal wind has no vertical structure.

For arbitrary *T*(*z*) and for general boundary conditions, the vertical structure Equation 1 for *L*_*n*_(*z*) is not a self‐adjoint Sturm–Liouville problem so a complete set of eigensolutions is not guaranteed to exist (and the eigenfunctions are not guaranteed to be orthogonal). However, it can always be solved numerically for any mean temperature profile *T*(*z*) as a boundary value problem subject to suitable boundary conditions, and the solutions yield the values of the separation constant, *h*_*n*_. Analytic solutions can be derived for an isothermal or piecewise isothermal (and similarly, for constant static stability, *N*, or piecewise uniform *N*; cf. Staniforth *et al*., 1985) atmosphere provided suitable combinations of boundary conditions are imposed at the lower and upper boundaries.

### 2.2 Mean temperature profiles

Equation 1 is solved in three atmospheric configurations: troposphere, combined troposphere‐stratosphere layer and a stratosphere‐only layer (the stratosphere‐only case is discussed in Appendix B). The mean temperature profiles substituted in Equation 1 in the first two atmospheric configurations are: constant temperature (the height‐weighted mean of the realistic temperature profile) and a “realistic” profile based on the US Standard Atmosphere (1976) that can be found also in textbooks, e.g. fig. 1.1 of Andrews *et al*. (1987). In the troposphere the realistic profile is very close to linear, and analytic solutions can be derived that are expressed as combinations of Bessel functions (see eq. 9.1.51 with *p* = 1 of Abramowitz and Stegun, 1972; Wiin‐Nielsen, 1971), but the analytic solutions do not provide insight here since the boundary conditions do not eliminate any of the Bessel functions. The constant temperature profile is studied here because an analytic solution can be derived for certain boundary conditions (that can also be compared with numerical solutions). For a realistic temperature profile, Equation 1 can only be solved numerically. In the third, stratosphere‐only, configuration (treated in Appendix B) we applied two additional temperature profiles: linear and parabolic approximations of the realistic profile. In the combined (troposphere‐stratosphere) configuration, the linear and parabolic profiles do not approximate the temperature variation.

### 2.3 Boundary conditions

The natural lower boundary condition at the ground is the vanishing of the vertical velocity, *w*. Using the thermodynamic energy equation and the gas law, *w* can be expressed as a linear combination of *L*_*n*_(*z*) and its derivative, *L*_*n*_'(*z*), so the condition *w* = 0 (i.e. a Robin boundary condition) implies $\frac{dL_{n}}{\mathrm{dz}}+\left(\frac{1}{h_{n}}-\frac{g}{\mathrm{RT}\;}\right)L_{n}=0$ (for more details see Lindzen and Chapman (1969) and Ch. 9 of Daley (1991), who derived the equations in pressure coordinates).

In the absence of a solid boundary, there is no natural choice for the upper boundary condition that determines the solutions for the eigenvalues *h*_*n*_ and the eigenfunctions *L*_*n*_. Thus, this upper boundary condition can be the vanishing of either *L*_*n*_(*z*) or *L*_*n*_′(*z*) (or a combination of *L*_*n*_(*z*) and *L*_*n*_′(*z*)), while the *w* = 0 condition is applicable only when the upper boundary is located at infinity. Despite its questionable relevance, the *w* = 0 condition was imposed in previous studies at some finite low‐pressure level (e.g. 4.3 hPa, as in Castanheira *et al*. (1999)). In the present study we solve Equation 1 subject to *w* = 0 at the bottom boundary (but in the stratosphere‐only configuration the vanishing of either *L*_*n*_(*z*) or *L*_*n*_′(*z*) are also considered) and for all three conditions: *L*_*n*_(*z*) = 0, *L*_*n*_′(*z*) = 0 or *w* = 0 at the top boundary (at finite height). The robustness of the results to the height of the upper boundary is also assessed. All combinations are solved numerically but in certain cases analytic or semi‐analytic solutions (see Section 3 below) can also be derived for a constant temperature profile.

At this point it should be noted that as mentioned above in the introduction, another form of boundary condition is often used in the absence of a rigid upper boundary – the radiation condition. This condition is often employed to eliminate one of the two known independent solutions of a 2nd‐order linear equation based, say, on the fact that waves (or energy) should propagate only away from (and not into) the domain since no source exists outside of it (see e.g. the discussion on p. 118 of Lindzen and Chapman (1969) and the survey in the introduction of Staniforth *et al*. (1985)). Detailed analyses (e.g. eqs 4.11 and 4.14 in Staniforth *et al*. (1985); eq. 2.10c‐d in Cohn and Dee (1989)) of the applicability of this condition to Equation 1 have shown that in a finite (but large) vertical domain, where the pressure tends to 0 at the upper boundary, the radiation condition is approximated by the vanishing of *L*_*n*_′(*z*). Thus, the *L*_*n*_′(*z*) = 0 condition at the upper boundary (which is employed here) approximates the radiation condition when the top is sufficiently high and the pressure is small (see results in Sections 3 and 5).

## 3 ANALYTIC AND SEMI‐ANALYTIC SOLUTIONS FOR CONSTANT TEMPERATURE PROFILE

In the case when $\frac{\mathrm{dT}}{\mathrm{dz}}=0$, that is, *T*(*z*) = *T*_0_ = constant, Equation 1 is a constant‐coefficient differential equation that can be solved analytically. Denoting *H*_0_ = *RT*_0_/*g*, the general solution of Equation 1 is:

(2) $$L_{n}\left(z\right)=e^{\frac{1}{2H_{0}}z}\left(Ae^{\frac{1}{2H_{0}}\left(\sqrt{1-\frac{4\kappa H_{0}}{h_{n}}}\right)z}+Be^{-\frac{1}{2H_{0}}\left(\sqrt{1-\frac{4\kappa H_{0}}{h_{n}}}\right)z}\right),$$

when *h*_*n*_ ≠ 4*κH*_0_, and

(3) $$L_{n}\left(z\right)=e^{\frac{1}{2H_{0}}z}\left(A+\mathrm{Bz}\right),$$

when *h*_*n*_ = 4*κH*_0_.

As in all linear and homogeneous eigenvalue problems, the boundary conditions determine the relation between the constants *A* and *B* in each of the solutions – Equations 2 and 3. The determination of this particular solution then leads to the determination of the eigenvalue *h*_n_.

### 3.1 Identical boundary conditions

For identical no‐normal‐flow top and bottom boundary conditions, the application of the boundary conditions (see above) $w=\frac{dL_{n}}{\mathrm{dz}}+\left(\frac{1}{h_{n}}-\frac{1}{H_{0}\;}\right)L_{n}=0$ to Equation 2 at *z* = *z*_bot_ and *z* = *z*_top_ yields (after tedious algebraic manipulations, as in Equation A1 in Appendix A below)

(4) $$\begin{array}{cc} & \underset{a}{\underbrace{\left({\left(\frac{1}{h_{n}}-\frac{1}{2H_{0}}\right)}^{2}-\frac{1}{{4H}_{0}^{2}}\left(1-\frac{4\kappa H_{0}}{h_{n}}\right)\right)}}\\ & \times \underset{b}{\underbrace{\left(e^{-\frac{\left(z_{\mathrm{top}}-z_{\mathrm{bot}}\right)}{2H_{0}}\left(\sqrt{1-\frac{4\kappa H_{0}}{h_{n}}}\right)}-e^{\frac{\left(z_{\mathrm{top}}-z_{\mathrm{bot}}\right)}{2H_{0}}\left(\sqrt{1-\frac{4\kappa H_{0}}{h_{n}}}\right)}\right)}}=0. \end{array}$$

This equation is satisfied when either *a* (the term in the top line) or *b* (the term on the LHS of the second line) equal 0 (or both). The solutions of Equation 4 are divided into two types (defined in e.g. Andrews *et al*., 1987; Daley, 1991): external modes that originate from *a* = 0 and internal modes that originate from *b* = 0. The equivalent depth of the single external mode (a.k.a. barotropic mode, e.g. Castanheira *et al*., 1999) denoted here by the subscript “baro”, is

(5) $$h_{\text{baro}}=\frac{H_{0}}{1-\kappa }=\frac{RT_{0}}{g\left(1-\kappa \right)},$$

and the associated vertical structure function, obtained by substituting this expression of *h*_baro_ into Equation 2 and applying the boundary condition *w*(*z*_bot_) = 0, is

(6) $$L_{\text{baro}}\left(z\right)=De^{\frac{\kappa }{H_{0}}\left(z-z_{\mathrm{bot}}\right)},$$

where *D* is an arbitrary amplitude.

The equivalent depth of this barotropic mode is independent of the layer thickness, Δ*z* = *z*_top_ − *z*_bot_. In previous studies this mode was denoted by the subscript “0” but here this designation is reserved for the baroclinic *n* = 0 mode studied in subsection 3.2 below. As is evident from Equation 6 the suggested definition of the barotropic mode based on *a* = 0 in Equation 4 does *not* imply vertical homogeneity of the vertical structure function.

The equivalent depths of the internal modes (a.k.a. baroclinic modes) are given by:

(7) $$h_{n}=\frac{4\kappa H_{0}}{1+{\left(\frac{2H_{0}\pi n}{\Delta z}\right)}^{2}},$$

where *n* = 1, 2, 3, … The associated vertical structure (obtained by substituting this expression of *h*_n_ into Equation 2, applying the boundary condition *w*(*z*_bot_) = 0 and implementing some more algebraic manipulations) has the form:

(8) $$\begin{array}{cc} & L_{n}\left(z\right)={De}^{\frac{1}{2H_{0}}z}\left(\cos\left(\frac{\pi n\left(z-z_{\mathrm{bot}}\right)}{\Delta z}\right)\right.\\ & \left.+\frac{\Delta z}{\pi nH_{0}}\left(\frac{1}{2}-\frac{{\left(2H_{0}\pi n\right)}^{2}+{\left(\Delta z\right)}^{2}}{4\kappa {\left(\Delta z\right)}^{2}}\right)\sin\left(\frac{\pi n\left(z-z_{\mathrm{bot}}\right)}{\Delta z}\right)\right), \end{array}$$

where *D* is an arbitrary amplitude.

Substituting *n* = 0 in Equation 7 yields *h*_*n*_ = 4*κH*_0_, which contradicts the condition *h*_*n*_ ≠ 4*κH*_0,_ under which Equation 2 was derived. In the particular case when *h*_*n*_ = 4*κH*_0_ the solution of Equation 1 is given by Equation 3 and applying the boundary conditions, *w* = 0 at *z*_bot_ and at *z*_top_, leads to the trivial solution, *L*_0_(*z*) = 0, so there is no *n* = 0 internal (baroclinic) mode when the boundary conditions are identical. Similar considerations eliminate the *h*_*n*_ = 4*κH*_0_ solution in the limit when Δ*z* → ∞.

### 3.2 Mixed boundary conditions

When the boundary conditions are mixed (i.e. the top and bottom conditions are *not* identical), there are no analytic solutions, but a semi‐analytic solution can be derived as follows: we first construct a matrix by substituting the general form of the solution in Equation 2 (including the derivatives of *L*_*n*_) into the two boundary conditions, and this matrix multiplies the vector of *A* and *B* coefficients (as in Equation A1 in Appendix A). For a non‐trivial solution, the determinant of this matrix must vanish. Straightforward algebraic calculations then yield a transcendental equation for some *x*(*h*_*n*_) (see below in Equations 9 and 10) which can be solved graphically by plotting both sides of this transcendental equation and finding the intersections between the curves to yield the values of *h*_*n*_.

In the two combinations involving mixed boundary conditions of *w* = 0 at the bottom and either *L*_*n*_ = 0 or *L*_*n*_' = 0 at the top of the layer, the resulting equation has the form

(9) $$\tan\left(x\right)=G\left(x\right)\cdot x$$

for *h*_*n*_ < 4*κH*_0_ where $x=\frac{\Delta z}{2H_{0}}\left(\sqrt{\frac{4\kappa H_{0}}{h_{n}}-1}\right)$ and *G*(*x*) is an expression determined separately for each of the specific combination of boundary conditions. An important property of *G*(*x*) is that it approaches a constant in either of the limits *x* → 0 or *x* → ∞. The expressions for *G*(*x*) are given in Appendix A.

For *h*_*n*_ > 4*κH*_0_ the equation obtained by substituting the general solution in the two boundary conditions and requiring that the determinant of this matrix vanishes is:

(10) $$\tanh\left(x\right)=G\left(x\right)\cdot x,$$

where $x=\frac{\Delta z}{2H_{0}}\left(\sqrt{1-\frac{4\kappa H_{0}}{h_{n}}}\right)$ and *G* has the same general form as in Equation 9 (see Appendix A).

In the boundary between these two regions, *h*_*n*_ = 4*κH*_0_, a dividing mode exists in a single combination of boundary conditions: *w*(*z*_bot_) = 0 and *L*(*z*_top_) = 0. These boundary conditions and *h*_*n*_ = 4*κH*_0_ yield a non‐trivial solution (analogous to the solution of Equation 3) provided $\Delta z=\frac{8}{3}H_{0}$.

Semi‐analytic (graphical) solutions of Equations 9 and 10 are calculated by the intersections of their two sides. Solutions of Equation 10 are the external (barotropic) modes and solutions of Equation 9 are the internal (baroclinic) modes and these solutions are counted according to the branch of the tangent function on the left‐hand side (LHS) of this equation (i.e. tan(*x*) is *nπ*‐periodic). Thus, the mode number is determined according to the eigenvalue and not necessarily by the number of zero‐crossings of the eigenfunction (only in cases where the differential problem has the structure of a Sturm–Liouville eigenvalue problem do the number of zero crossings equal the mode number, see Figure 8 below). Clearly, since the slope of the tan(*x*) function is 1 near *x* = 0 and increases with *x*, the RHS of Equation 9 does not intersect the first (i.e. *n* = 0) branch of the tan(*x*) function when 0 < *G*(*x*) < 1 near *x* = 0. This restriction implies that no *n* = 0 mode exists for small $\frac{2H_{0}}{\Delta z},$ i.e. in thick layers or for low mean temperatures. Similarly, the slope of the tanh(*x*) function near *x* = 0 is also 1 and decreases with *x*, and therefore the similar considerations employed for the *n* = 0 baroclinic mode apply also to the barotropic modes of Equation 10 when *G*(*x*) > 1. Note that for *G*(*x*) < 1 a solution of Equation 10 can exist at large *x* only for *h*_*n*_ > Δ*z*, that is, when the equivalent depth is larger than the layer's thickness. In addition, since only positive values of the first branch of tan(*x*) and tanh(*x*) yield acceptable solutions, cases with *G*(*x*) < 0 for all *x* cannot be associated with solutions.

The (eigen)value of *h*_*n*_ (determined as described above from the solutions for *x*) is substituted in the general form of the solution, Equation 2, to yield the corresponding eigenfunction of each eigenvalue. As in the case of identical boundary conditions, when the boundary conditions are mixed, the eigenfunctions are also combinations of sine and cosine functions for *h*_*n*_ < 4*κH*_0_ and combinations of growing and decaying exponents for *h*_*n*_ > 4*κH*_0_.

The analytic and semi‐analytic solutions presented in this section provide an anchor for the interpreting the numerical solutions to be presented next.

## 4 METHODS OF NUMERICAL SOLUTION

For non‐constant temperature profiles, only numerical solutions will be computed. The actual values of *h*_*n*_ and the corresponding eigenfunctions will be computed in Section 5 for the two temperature profiles (and in Appendix B for the four stratospheric temperature profiles). The identification of the various modes (barotropic, *n* = 0 baroclinic mode and *n* ≥ 1 baroclinic modes) is made based on a comparison between the structure of the eigenfunctions computed numerically and those of the eigenfunctions of the constant temperature profile solved analytically (or semi‐analytically). In all cases, a double check of the proximity of the *h*_*n*_ (eigen)values to those of the constant temperature profile is used to confirm the mode identification.

The vertical Equation 1 for *L*_*n*_(*z*) is solved numerically (including the value of *h*_*n*_) using two different methods of solution: the shooting method and the Chebyshev spectral collocation method. A brief description of these methods is provided in this section.

### 4.1 The shooting method

In the shooting method a differential set that contains an unknown eigenvalue is integrated from one boundary, starting with the boundary condition that applies to this boundary (including possibly a normalization condition), to the other boundary. The boundary condition at the other boundary is then used as a “cost function” that is minimized (vanishes) for discrete values of the unknown eigenvalue. Alternatively, the set can be integrated numerically from each of the boundaries to an inner matching point at which the continuity of the two variables is the “cost function” that determines the values of the eigenvalue. The shooting method has been successfully applied in problems with complex eigenvalues (e.g. Killworth *et al*., 1984), problems with singularities (e.g. Paldor and Dvorkin, 2006) and in problems with real eigenvalues (Cohen *et al*., 2016). Here we apply this method to solve the vertical Equation 1 with the temperature profiles and boundary conditions detailed in subsections 2.2 and 2.3.

### 4.2 Chebyshev spectral collocation method

In the second, independent, spectral method of Chebyshev collocation the domain is divided into a grid of discrete (collocation) points at which the solution is given by the eigenvector of the algebraic eigenvalue (matrix) problem obtained when the differential operators (as well as the coefficient functions) are approximated by their finite difference counterparts. The values of the eigenfunctions at the points between the collocation points are approximated by a high‐order Chebyshev polynomial and eigenvalues of the algebraic system approximate those of the differential set. A general description of the details of this method, including the application of the boundary conditions, is given in Trefethen (2000) and Poulin and Flierl (2003). The method was successfully applied to solve eigenvalue problems associated with the shallow‐water equations in various geometries (e.g. De‐Leon and Paldor, 2009; 2011). Here, this method is applied to solve the second‐order vertical Equation 1 in the atmosphere for two temperature profiles (constant and realistic) when *L*_*n*_(*z*) vanishes at both lower and upper boundaries (this case is treated in Appendix A).

The numerical results are confirmed by comparing the solutions obtained by the two (independent) numerical methods with each other. In cases where analytic solutions of Equation 1 exist, that is, for constant temperature profile and for certain boundary conditions, the numerical solutions are compared with the analytic solutions or with the semi‐analytic (graphic) solutions detailed in Section 3, and as discussed below are accurate to, for example, at least the fourth significant digit.

## 5 RESULTS

We now present results on the sensitivity of the eigenfunctions and eigenvalues (i.e. equivalent depths) of Equation 1 to the temperature profile and boundary conditions both for a troposphere‐only configuration and for a configuration with both a troposphere and stratosphere.

### 5.1 Troposphere

We solve Equation 1 numerically for two temperature profiles: (a) a realistic (linear) profile based on the US Standard Atmosphere (1976), (b) a constant profile with *T*(*z*) = *T*_0_ = 254 K, that is, the (height‐)weighted mean of the realistic profile that varies between 290 and 218 K. The relevant boundary conditions in the troposphere are those with *w* = 0 at the bottom and *L*_*n*_ = 0 or *L*_*n*_′ = 0 at the top. We consider also the case of *w* = 0 at both bottom and top though it is not realistic in the troposphere (see subsection 2.3) because we wish to compare these results with previous studies and since this case has an exact analytic solution (for constant temperature), which can validate the accuracy of the numerical calculations. In Appendix A we extend our analysis to all nine combinations of boundary conditions (*L*_*n*_ = 0, *L*_*n*_′ = 0 or *w* = 0 at both bottom and top) to better understand the classification of the different modes into external (barotropic) and internal (baroclinic).

For constant temperature profile, there is an excellent agreement between the analytic or semi‐analytic and numerical solutions. For identical boundary conditions and according to Equation 5, the equivalent depth of the external (barotropic) mode is *h*_baro_ = 10,403 m and an identical value was also obtained in our numerical calculations. Similarly, according to Equation 7 the analytic value of the first internal (baroclinic) mode (i.e. *n* = 1 since no *n* = 0 baroclinic mode exists when the top and bottom boundary conditions are identical) is *h*_1_ = 447 m and this is precisely the value obtained in our numerical solutions. These excellent agreements validate the accuracy of our numerical solutions.

**Figure 1:**
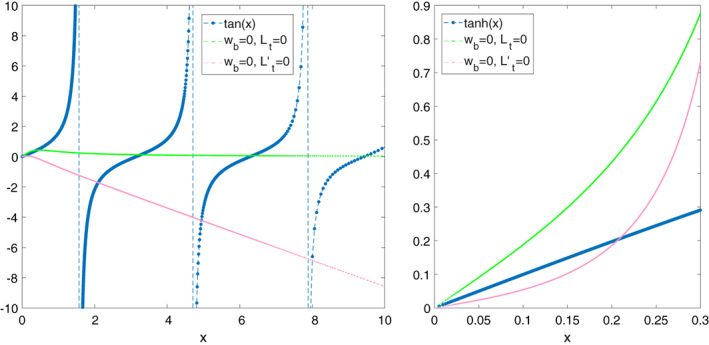
The two sides of Equations 9 (left panel) and 10 (right panel) in the troposphere. Dotted blue lines mark the left‐hand side of these equations and the coloured lines are the right‐hand side of these equations for different combinations of boundary conditions. Only *x* > 0 values are considered since *z*_top_ > *z*_bot_ implies *x* > 0 and in the right panel only positive right‐hand side curves were plotted, since a negative curve would not intersect the (positive) tanh(*x*). Subscripts b and t denote bottom and top, respectively

For the two combinations of mixed boundary conditions (*w* = 0 at the bottom and *L*_*n*_ = 0 or *L*_*n*_′ = 0 at the top), only semi‐analytic solutions could be derived, and Figure 1 shows that these semi‐analytic solutions agree with the numerical solutions to within 10^−4^. The left panel of Figure 1 shows the intersections between the two sides of Equation 9 while the right panel shows one intersection of both sides of Equation 10. In the right panel, the two combinations of mixed boundary conditions have positive values of the RHS of Equation 10 at small *x* (and can intersect the positive half of the tanh(*x*) function) but intersection with the tanh(*x*) occurs only for one of the combinations and this single intersection yields an equivalent depth which is very close to that of the analytic barotropic mode. In the left panel, only one combination of mixed boundary conditions has positive values of the RHS of Equation 9 at small *x* and intersects the positive half of the first, that is, the *n* = 0 branch of the tangent function (recall: no *x* < 0 solutions), which yields the equivalent depth of the *n* = 0 baroclinic mode. The two intersections in each of the *n* ≥ 1 branches of the tangent function yield the equivalent depths of the *n* ≥ 1 baroclinic modes, where the second branch yields the *n* = 1 baroclinic mode whose eigenvalues are of the same order as the analytic *h*_1_ value.

**Figure 2:**
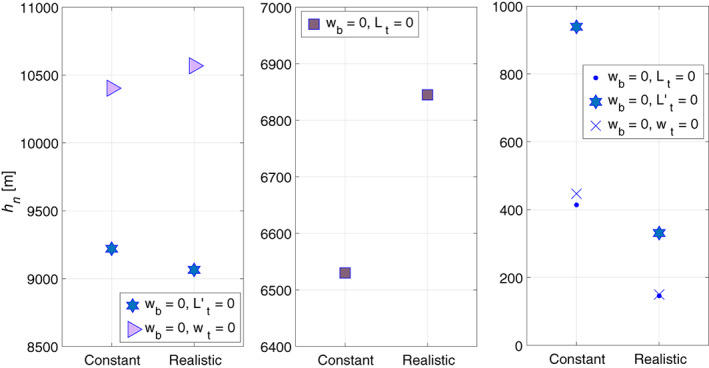
Equivalent depths in the troposphere in two temperature profiles – constant (left column in each panel) and realistic (right column in each panel) for the barotropic mode (left panel), the *n* = 0 baroclinic mode (middle panel) and the *n* = 1 baroclinic mode (right panel). Note that the scales of the ordinates differ widely between panels and that some of the symbols in the right panel overlap

Having solved Equations 9 and 10 for constant temperature profile we now turn to the solutions of Equation 1 for the realistic (i.e. linear in the troposphere) temperature profile. Our numerical solutions of Equation 1 for a realistic temperature profile have the same modal structure as the analytic, or semi‐analytic, modes of the constant temperature profile (results not shown). For barotropic modes the equivalent depths are similar to those of the constant profile (left panel of Figure 2) and these values are close to the analytic value of 10,403 m calculated for the constant profile. In the baroclinic modes the equivalent depths of the realistic profile differ by a factor of up to 3 from those of the constant profile. The equivalent depth of the *n* = 0 baroclinic mode (according to the intersection with the first branch of the tangent function on the left panel of Figure 1) is shown in the middle panel of Figure 2 and the *n* = 1 equivalent depths (for various combinations of boundary conditions) on the right panel of Figure 2. As could be expected, for all boundary conditions and temperature profiles the equivalent depths of the *n* > 0 baroclinic modes are smaller than those of the *n* = 0 baroclinic mode which are smaller than those of the barotropic mode.

**Figure 3:**
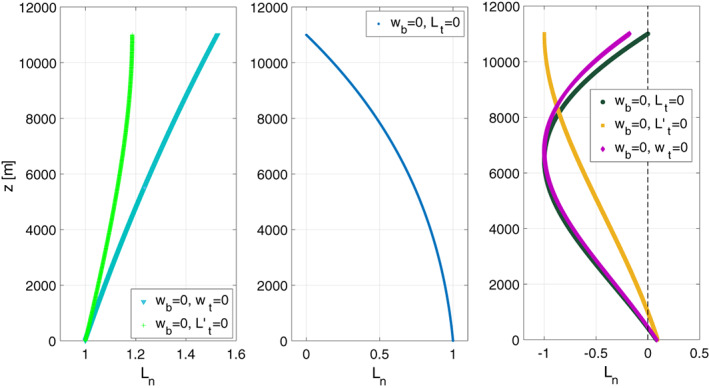
Eigenfunctions in the troposphere of the barotropic mode (left panel), *n* = 0 baroclinic mode (middle panel) and *n* = 1 baroclinic mode (right panel), all for constant temperature profile. The functions are normalized such that the minimum is 1 in the left panel, −1 in the right panel and the maximum is 1 in the middle panel

Having found the solutions for the eigenvalues, *h*_*n*_, we turn our attention to the corresponding eigenfunctions and the sensitivity of the solutions to the mean temperature profiles and boundary conditions. Figure 3 shows the corresponding eigenfunctions, *L*_*n*_(*z*), for constant temperature where the numerical solutions are identical (up to the width of the line) with the analytic solutions of Equations 6 and 8, and with the eigenfunctions of the cases with mixed boundary conditions, when the values of *h*_*n*_ obtained from the intersections of Figure 1 are used. The left panel of Figure 3 shows the barotropic mode, determined by both the value of the associated equivalent depth and by the monotonic increase of the eigenfunction in the lower few kilometres, which is nearly identical with the analytic eigenfunction of the barotropic mode, Equation 6. The middle panel shows the *n* = 0 baroclinic mode (associated with the *n* = 0 baroclinic equivalent depth of Figure 2) and the right panel shows the *n* = 1 baroclinic modes. The functional form of the *n* ≥ 1 baroclinic modes depends strongly on the upper boundary condition.

The eigenfunctions, *L*_*n*_, for a realistic temperature profile, are nearly identical to those of the constant temperature profile (results not shown).

The conclusions from these results are that in the troposphere: (a) the barotropic mode can only exist when *w* = 0 at the bottom and for realistic values of the ratio between the temperature and the layer thickness (i.e. $\frac{2H_{0}}{\Delta z}$); (b) the temperature profile has a minor effect on the value of the equivalent depth of barotropic modes; and (c) baroclinic modes exist in all boundary condition combinations, and their associated equivalent depths vary with the temperature profile but their vertical structure is almost unaffected by the temperature profile. The first row of Table 1 summarizes the existence of the various modes in the troposphere for different combinations of boundary conditions.

**Table 1:** Summary of the relevant top boundary condition that permit the existence of the various modes in the troposphere and the combined troposphere‐stratosphere layer when the boundary condition at the bottom is *w* = 0

	Barotropic mode	*n* = 0 baroclinic mode	*n* ≥ 1 baroclinic mode
Troposphere	*L* ′ (*z*_top_) = 0 *w*(*z*_top_) = 0	*L*(*z*_top_) = 0	*L*(*z*_top_) = 0 *L* ′ (*z*_top_) = 0 *w*(*z*_top_) = 0
Combined troposphere‐stratosphere	*L*(*z*_top_) = 0 *L* ′ (*z*_top_) = 0 *w*(*z*_top_) = 0	No	*L*(*z*_top_) = 0 *L* ′ (*z*_top_) = 0 *w*(*z*_top_) = 0

### 5.2 Combined troposphere‐stratosphere

We solve Equation 1 numerically for two temperature profiles: (a) a realistic profile based on the US Standard Atmosphere (1976); and (b) a constant profile with *T*(*z*) = *T*_0_ = 248 K – the (height‐)weighted mean of the realistic profile that varies between 290 and 218 K. As in the troposphere, the relevant realistic boundary conditions in the combined troposphere‐stratosphere layer are *w*(*z*_bot_) = 0 and *L*_*n*_ = 0 or *L*_*n*_' = 0 at the top, but we extend our analysis also to the case with *w* = 0 at the top.

For constant temperature profile, and as in the troposphere, the agreement is excellent between the analytic (or semi‐analytic) and numerical solutions. No baroclinic *n* = 0 mode exists (i.e. no intersections of the RHS of Equation 9 with the first branch of the tan(*x*) function). Also, there are two intersections of the RHS of Equation 10 with the tanh(*x*) function (results not shown), that yield *h*_*n*_ values which are relevant and close to the analytic barotropic mode, and associated eigenfunction which is similar to that of the analytic barotropic mode.

**Figure 4:**
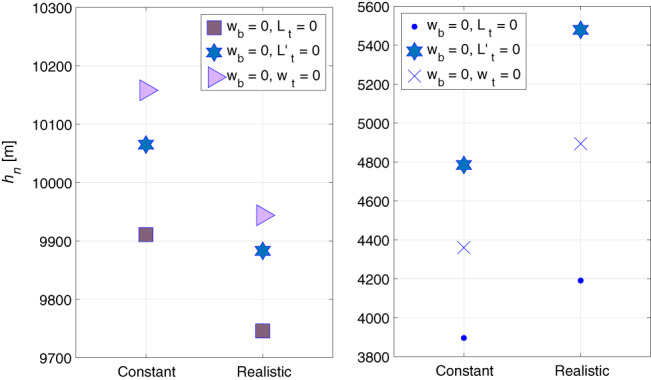
Equivalent depths in the combined troposphere‐stratosphere layer in two temperature profiles – constant (left column in each panel) and realistic (right column in each panel) for the barotropic mode (left panel) and the *n* = 1 baroclinic mode (right panel). Note that the ordinate scales differ between the two

For both constant and realistic temperature profiles, Figure 4 compares the equivalent depths for different combinations of boundary conditions. The left panel shows the equivalent depths of the barotropic mode and the right panel shows the *n* = 1 baroclinic modes. The values of barotropic equivalent depths on the left panel (9950 ± 200 m, depending on the imposed boundary conditions) are very similar to each other and of the same order as the analytic value of 10,158 m calculated (analytically and numerically) for constant temperature (the slight difference between this analytic value of 10,158 m and the tropospheric value (10,403 m) originates from the different mean temperature used). The equivalent depths of the *n* = 1 baroclinic mode are *h*_1_ = 4700 ± 800 m (depending on the imposed boundary conditions) and are similar to the analytic solution of 4,360 m of the combination of identical boundary conditions for which an analytic solution is derived for constant temperature.

The structures of the eigenfunctions in this combined layer are very similar to those of the troposphere (up to relevant scaling) so the results are not shown. The effect of the temperature profile on these eigenfunctions (as well as on the equivalent depths, see Figure 4) is negligible.

The conclusions from the above are that in the combined troposphere‐stratosphere layer: (a) the barotropic mode exists when *w* = 0 at the bottom, no *n* = 0 baroclinic mode exists, and *n* ≥ 1 baroclinic modes exist in all boundary condition combinations; and (b) the equivalent depths of both barotropic and baroclinic modes are insensitive to the details of the temperature profile while those of the baroclinic modes are sensitive to the boundary conditions imposed. The second row of Table 1 summarizes the existence of the various modes in the combined troposphere‐stratosphere layer for different combinations of boundary conditions.

### 5.3 Sensitivity of the solutions to layer thickness

**Figure 5:**
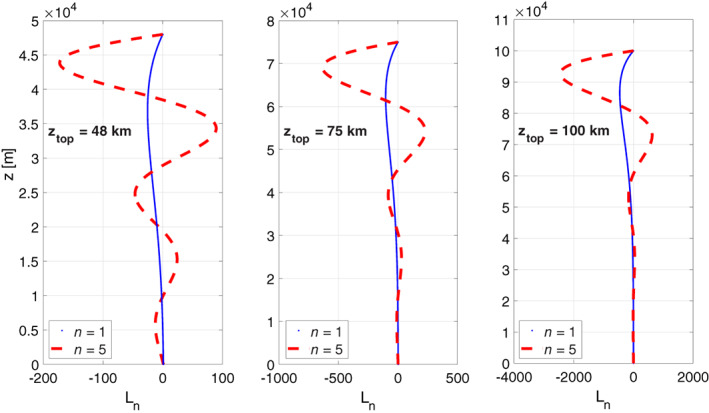
Eigenfunctions of the *n* = 1 baroclinic mode (solid blue) and *n* = 5 baroclinic mode (dashed red line), for different layer thicknesses (48 km – left panel, 75 km – middle panel, 100 km – right panel) all for constant temperature profile

**Figure 8:**
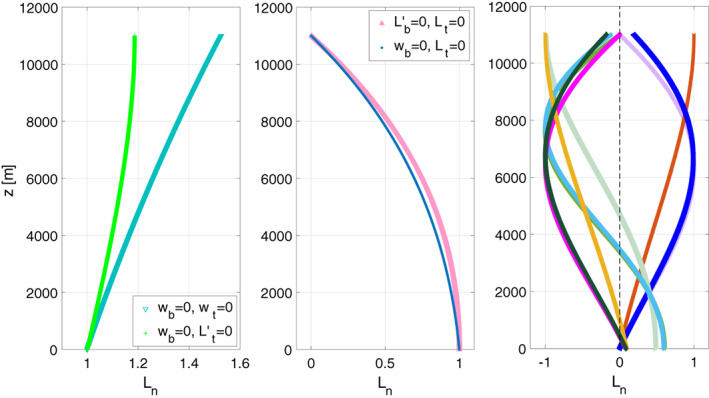
Eigenfunctions in the troposphere of the barotropic mode (left panel), *n* = 0 baroclinic mode (middle panel) and *n* = 1 baroclinic mode (right panel), all for constant temperature profile. The functions are normalized such that the minimum value of the function is −1 or 1 or that the maximum value is 1. Note that some of the curves in the right panel do not cross the zero line even once, even though they are the eigenfunctions of the *n* = 1 baroclinic mode

We have explored the sensitivity to increasing the layer thickness of the combined troposphere‐stratosphere layer by repeating the calculation but for *z*_top_ = 75 km and *z*_top_ = 100 km. The effect of this increase was examined for the constant temperature profile as well as for the realistic profile (which was extended linearly up to *z*_top_) and for various combinations of boundary conditions. As a representative example, Figure 5 shows the eigenfunctions of the *n* = 1 and *n* = 5 baroclinic modes for three different values of *z*_top_ for constant temperature and for the boundary condition combination of *w*(*z*_bot_) = 0 and *L*(*z*_top_) = 0. Two dependencies of the eigenfunctions on *z*_*top*_ are evident in Figure 5: first, the eigenfunctions are stretched in the vertical, and second, the maximum amplitude of the eigenfunctions (attained at or near *z*_top_) increases with *z*_top_. The vertical stretching leads to differences in the heights at which the eigenfunction's zero crossings occur. However, for the barotropic mode or baroclinic modes without zero crossings, the overall structure of the eigenfunctions is not sensitive to the height of the top boundary. For realistic temperature profile and/or for other combinations of boundary conditions the results are similar and not shown.

The equivalent depths increased by a factor of 2 to 4 when the layer thickness was doubled (results not shown). For constant temperature profile the (nonlinear) increase in *h*_*n*_ is evident from Equation 7 where an increase of Δ*z* decreases the denominator so *h*_*n*_ increases. Substituting typical values of *T*(*z*) = *T*_0_ = 240 K and *κ* = 2/7 in Equation 7 yields $h_{n}=\frac{4\kappa H_{0}}{1+{\left(\frac{2H_{0}\pi n}{\Delta z}\right)}^{2}}=\frac{8\cdot 10^{3}}{1+{\left(\frac{44\cdot 10^{3}\cdot n}{\Delta z}\right)}^{2}}$, so for the *n* = 1 baroclinic mode and for small values of Δ*z* ≪ 44 · 10^3^ m, the second term in the denominator is much larger than 1 so *h*_*n*_ is nearly parabolic in Δ*z*. For large values of Δ*z* ≫ 44 · 10^3^ m, the second term of the denominator is neglected compared to 1 and *h*_*n*_ approaches a constant asymptotic value of ∼8 km. For n > 1 baroclinic modes, the variation of *h*_*n*_ with Δ*z* is slower. For realistic values of Δ*z* that vary between 10 km in the troposphere and 50 km in the combined troposphere‐stratosphere, *h*_*n*_ increases monotonically with Δ*z* (parabolically when Δ*z* ≪ 44 · 10^3^ m).

Overall, the part of the eigenfunction within the stratosphere for modes with no zero crossings is little changed when the upper boundary is moved from the stratopause into the mesosphere. The equivalent depths (i.e. eigenvalues) do change as the top boundary is moved into the mesosphere, though the nature of the change can be accounted for using the theory described in Section 3.

## 6 SUMMARY AND DISCUSSION

The phase speed and structure of wave solutions of the primitive equations in both the horizontal and vertical directions depend critically on the equivalent depth. While in some cases the equivalent depth can be deduced from the forcing that initially launches the wave, for a more general free mode the equivalent depth must be calculated from an eigenanalysis of the vertical structure equation. In this work we analyse how the eigenanalysis of the vertical structure equation (for two atmospheric configurations) depends on the boundary conditions imposed, the temperature profile about which one solves, and the height at which the upper boundary is imposed.

Having obtained the value of the equivalent depth (*h*_*n*_), we can substitute it in the horizontal equations (on either plane or sphere) to get the meridional structure of the horizontal waves and the associated frequencies. This application of the vertical solutions found here to the calculation of the horizontal modes of the shallow‐water equations is left to future work.

The addition of a mean zonal flow that depends on height to the primitive equations prevents the separation of the vertical and horizontal equations. Mathematically, separation is possible only when $\bar{p}\left(y,z\right)=\bar{p_{1}}\left(y\right)+\bar{p_{2}}\left(z\right)$, while $\bar{T}\left(y,z\right)=\bar{T_{2}}\left(z\right)$ and $\bar{u}\left(y,z\right)=\bar{u_{1}}\left(y\right)$. The reason for this is that in the presence of a mean zonal flow, $\bar{u}$, the geostrophic balance, $f\bar{u}=-\frac{1}{\rho }\frac{\partial \bar{p}}{\partial y}$, mandates that the mean pressure, $\bar{p}$, is *y*‐dependent, that is, $f\bar{u_{1}}=-\frac{1}{\rho }\frac{\partial \bar{p_{1}}}{\partial y}$. On the other hand, the hypsometric equation, $\frac{\partial \ln\bar{p}}{\partial z}=-\frac{g}{R\bar{T}}$, mandates that if the mean temperature, $\bar{T}$, varies with *z* so does the pressure, that is, $\frac{\partial \ln{\bar{p}}_{2}}{\partial z}=-\frac{g}{R\bar{T_{2}}}$. If these two relations are associated with distinct (i.e. additive and not multiplicative) pressure contributions, then separability is possible (e.g. Winter and Schmitz, 1998). As this article relies on the separability of the vertical and horizontal equations, all results in this article are only valid if the zonal wind, $\bar{u}$, depends on latitude only or is constant.

The main findings of this work are:

- For constant mean temperature profiles (where analytic or semi‐analytic solutions exist):
  1. The exponential form of the barotropic mode can be derived analytically for identical top and bottom boundary conditions. In mixed boundary conditions it is derived by the single intersection of a linear function with a *hyperbolic tangent* function.
  2. In contrast, baroclinic modes oscillate with height (and can also be derived analytically for identical top and bottom boundary conditions) and for mixed boundary conditions they are obtained by the intersection of a linear function with a *tangent* function (hence there exist a countable number of modes).
  3. When the slope of the linear function is smaller than one, it does not intersect the first branch of the tangent function (whose minimal slope of 1 occurs at the origin) and so no *n* = 0 baroclinic mode exists.
- For other mean temperature profiles (where only numerical solutions can be derived): very similar properties to those outlined in a. are encountered numerically, even though the problem is not Sturm–Liouville.

Surprisingly, for an isothermal atmosphere, the solutions of *h*_*n*_(*x*) for all combinations of mixed boundary conditions can be described by Equation 9 (with slightly different *G*(*x*)) for baroclinic modes (including those detailed in Appendix B), or by Equation 10 for barotropic modes. Thus, the general procedure for finding the semi‐analytic solutions is applicable to all modes and boundary conditions.

No *n* = 0 baroclinic mode exists in the combined troposphere‐stratosphere layer since this layer is too thick for the RHS of Equation 9 to intersect the first branch of the tan(*x*) at some *x* ≠ 0, i.e. *G*(0) is too small. For the combination of boundary conditions of *w*(*z*_bot_) = 0 and *L*(*z*_top_) = 0, the value of $G\left(x\to 0\right)=\frac{4}{3}\frac{2H_{0}}{\Delta z}$, and since in this limit Equation 9 is approximated by tan(*x*) = *G*(0) · *x* a solution of this equation occurs only when G(0) > 1, that is, a solution can only exist if $\Delta z<\frac{8}{3}H_{0}$. Thus, for a constant temperature of ∼250 K the layer thickness must be smaller than about 19.5 km for the baroclinic *n* = 0 mode to exist. Baroclinic modes with *n* ≥ 1 always exist regardless of Δ*z*.

The magnitudes of the equivalent depths in each layer are (much) smaller than the actual thickness of the layer. This is not surprising and was reported in previous studies such as Lindzen and Chapman (1969) and Kiladis *et al*. (2009). However, the corresponding values of $\sqrt{gh_{n}}$ calculated in the present study are quite large: ∼300 m·s^−1^ for the barotropic mode, 50–100 m·s^−1^ for the *n* = 1 baroclinic mode in the troposphere, and ∼200 m·s^−1^ for the *n* = 1 baroclinic mode in the combined troposphere‐stratosphere layer. The estimates for the baroclinic modes are larger than those reported by Kiladis *et al*. (2009) for convectively coupled equatorial waves. This difference in the equivalent depth can result from: (a) the neglect of moisture and diabatic heating; (b) the assumptions made in deriving the linearized primitive equations (i.e. nonlinear terms are neglected); or (c) the neglect of meridional temperature gradients.

It is quite remarkable that the calculated equivalent depths differ by less than ±50% for all combinations of boundary conditions and mean temperature profiles examined for a given configuration; that is, the choice of boundary condition and the details of the mean temperature profile have a small effect on the value of the equivalent depth. The small effect of the temperature profile results from the small relative change in the temperature (in Kelvin degrees, the total variation does not exceed ∼35 K out of ∼250 K), while the sensitivity to boundary conditions is more significant. The details of the assumed temperature profile are also relatively unimportant for higher baroclinic modes, as both equivalent depths and eigenfunctions are nearly identical for different temperature profiles. As in classical linear eigenvalue problems, the number of zero crossings of the eigenfunctions calculated here increases by 1 when the mode number increases by 1, for all temperature profiles even when the differential problem is not Sturm–Liouville (results not shown).

The equivalent depth for the barotropic mode is independent of the height at the upper boundary. The equivalent depth does increase for all other modes as the height of the upper boundary is increased, however, and hence the phase speed of free modes supported by, for example, a general circulation model are likely dependent on the location of the upper boundary, unless an additional process is present that leads to the vertical confinement of wave activity. When the height of the upper boundary is increased, the eigenfunctions are stretched in the vertical, but their characteristics are otherwise unchanged for modes with no zero crossings. For modes with zero crossings, the location of zero crossings is sensitive to the height of the upper boundary. Overall, imposing a lid too low in a region where for example, planetary Rossby waves are present will lead to a distortion of the modal structure and to a too‐slow phase speed, and therefore to an incorrect interaction of the wave with the mean flow.

These results also have implications for the role of resonance in the dynamics of sudden stratospheric warmings; see Appendix B for details.

As was mentioned in the introduction, the radiation boundary condition was employed as the upper boundary condition in many analytic (where the eigenvalues are known from the horizontal equations) or numerical studies of atmospheric dynamics. However, it cannot be applied here since it only eliminates one of two independent solutions of the second‐order, linear, differential vertical structure equation but cannot determine the eigenvalues (i.e. the equivalent depths) so its application leads to the conclusion that there are no vertical eigenmodes in the force‐free oscillations in the atmosphere (see e.g. Section 3 of Kasahara (1976)). For that reason, many studies used instead the upper boundary condition of vanishing *w* at z = *z*_top_ at some large *z*_top_ (e.g. Kasahara, 1976; Staniforth *et al*., 1985; Cohn and Dee, 1989; Daley, 1991; Castanheira *et al*., 1999). In addition, as is evident from Cohn and Dee (1989) and from the actual upper boundary condition used in Staniforth *et al*. (1985)'s analysis, the best approximant of the radiation condition at a finite, but small, pressure level, for example the stratopause, is the vanishing of the derivative of the sought eigenfunction (i.e. *L*_*n*_'(*z*_top_) = 0) which was also examined here.

The clear definitions of the barotropic modes, the *n* = 0 baroclinic mode (in mixed boundary conditions) and the higher baroclinic modes (a definition that is ill‐defined in the existing literature) can only be derived by imposing an upper boundary condition that suits the relevant boundary eigenvalue problem. (The same is true for the lower boundary condition of the “stratosphere only” case studied in Appendix B.) Such suitable boundary conditions are: Neumann, Dirichlet or Robin conditions but not the radiation condition which is commonly employed in the solution of the initial value problem, that is, in simulations by GCMs. Though the exact values of the equivalent depth vary somewhat with the combination of boundary conditions imposed, the typical value of O(10 km) (irrespective of Δ*z* – the layer thickness) of the barotropic mode is well separated from the significantly lower values of the baroclinic modes (that vary with Δ*z*). The equivalent depth of the *n* = 0 baroclinic mode is less than 6–7 km (and it varies slightly with the choice of boundary conditions) when it exists (i.e. when Δ*z* < 19.5 km). The equivalent depth of the high (i.e. *n* > 0) baroclinic modes is O(4 km) in the combined troposphere‐stratosphere layer and merely O(200 m) in the troposphere.

Our results regarding the relationship between *h*_*n*_ and Δ*z* are also relevant to baroclinic modes in the tropical atmosphere. Namely, for a given mode number, *n*, the equivalent depth is lower in the troposphere than in the combined troposphere‐stratosphere layer. Hence, a free mode generated *in situ* in the stratosphere will have a higher equivalent depth (and larger vertical wavelength) than a free wave generated in the troposphere. Similar results of an increase of the equivalent depth above the tropopause (relative to its value in the troposphere) were also reported by Kim *et al*. (2019) who compared equatorial wave baroclinic modes in the tropical tropopause layer and stratosphere in reanalysis data. Salby and Garcia (Garcia and Salby, 1987) find a similar effect in a linearized primitive equation model forced by convective‐like diabatic heating in the troposphere, and attribute it to stratospheric radiative damping preferentially acting on wave components with short vertical wavelength and small frequency, leaving behind higher‐frequency modes with larger vertical wavelengths as one moves higher in the stratosphere. While the overall result is the same – equivalent depths in the stratosphere are larger than in the troposphere – our mechanism is different from that of Garcia and Salby (1987), as our mechanism depends on *in situ* stratospheric generation while their mechanism assumes no stratospheric source.

The relative importance of these two mechanisms for the equivalent depth of observed waves in the stratosphere should be explored in future work.

## Figures and Tables

**Figure 6:**
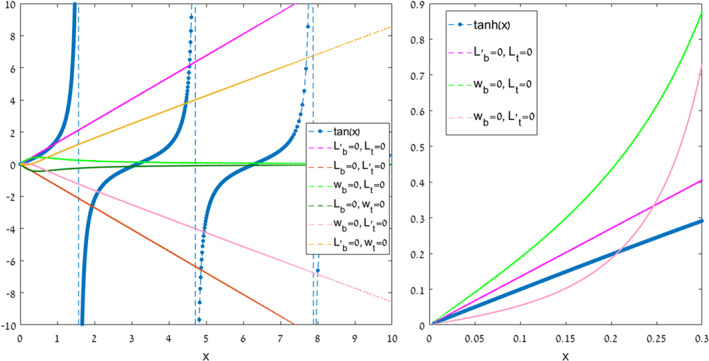
The two sides of Equations 9 (left panel) and 10 (right panel) in the troposphere. Dotted blue lines mark the left‐hand side of these equations and the coloured lines are the right‐hand side of these equations for different combinations of boundary conditions

**Figure 7:**
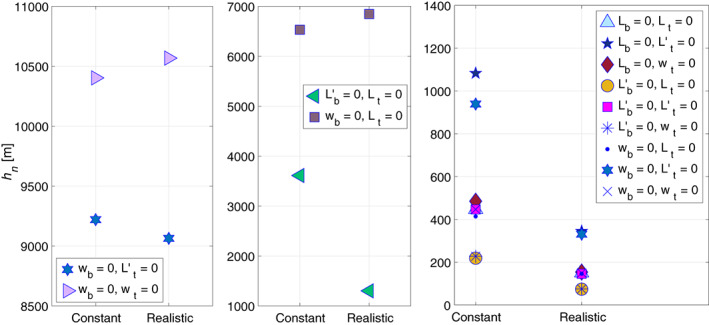
Equivalent depths in the troposphere in two temperature profiles – constant (left column in each panel) and realistic (right column in each panel) for the barotropic mode (left panel), the *n* = 0 baroclinic mode (middle panel) and the *n* = 1 baroclinic mode (right panel). Note that the scales of the ordinates differ widely between panels and that some of the symbols in the right panel overlap

**Figure 9:**
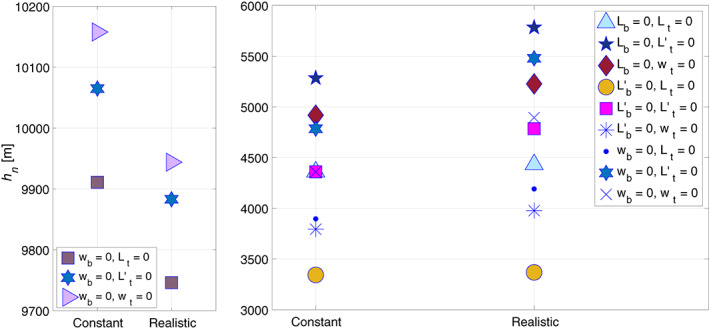
Equivalent depths in the combined troposphere‐stratosphere layer in two temperature profiles – constant (left column in each panel) and realistic (right column in each panel) for the barotropic mode (left panel) and the *n* = 1 baroclinic mode (right panel). Note that the ordinate scales differ between the two panels and that some of the symbols in the right panel overlap

**Figure B1:**
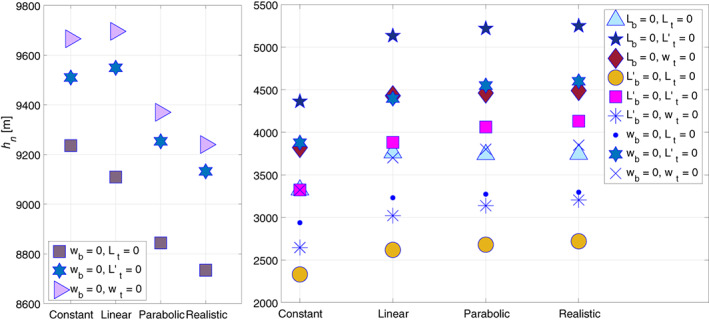
**FIGURE B1** The values of equivalent depths in the stratosphere for different combinations of boundary conditions and different temperature profiles, for the barotropic mode (which does not actually exist in the stratosphere since the *w* = 0 boundary condition is irrelevant to the tropopause; left panel) and the *n* = 1 baroclinic mode (right panel). Note that the scales of the ordinates differ widely between panels and that some of the symbols in the right panel overlap

## Appendix

For better understanding of the effect of boundary conditions on the eigenvalues, we examine here numerical solutions to Equation 1 upon applying all nine combinations of Dirichlet, Neumann and *w* = 0 at both bottom and top of each of the atmospheric set‐ups.

## 1 Identical boundary conditions

In the case when the top and bottom identical boundary conditions are *L*_*n*_(*z*_bot_) = 0 = *L*_*n*_(*z*_top_) the application of these boundary conditions to the explicit expression for *L*_*n*_(*z*) in Equation 2 is written in matrix form as:

(A1) $$\left[\begin{array}{cc} e^{\frac{z_{\mathrm{bot}}}{2H_{0}}\left(\sqrt{1-\frac{4\kappa H_{0}}{h_{n}}}\right)} & e^{-\frac{z_{\mathrm{bot}}}{2H_{0}}\left(\sqrt{1-\frac{4\kappa H_{0}}{h_{n}}}\right)}\\ e^{\frac{z_{\mathrm{top}}}{2H_{0}}\left(\sqrt{1-\frac{4\kappa H_{0}}{h_{n}}}\right)} & e^{-\frac{z_{\mathrm{top}}}{2H_{0}}\left(\sqrt{1-\frac{4\kappa H_{0}}{h_{n}}}\right)} \end{array}\right]\left[\begin{array}{c} A\\ B \end{array}\right]=0.$$

In order to guarantee that *A* and *B* are not both zero (in which case *L*_*n*_(*z*) vanishes identically) the determinant of the matrix on the LHS of Equation A1 has to vanish. After some additional algebraic manipulations, the vanishing of this determinant yields the expression for the internal modes (no external, barotropic, mode exists in this case):

(A2) $$L_{n}\left(z\right)=De^{\frac{1}{2H_{0}}z}\sin\left(\frac{\pi n\left(z-z_{\mathrm{bot}}\right)}{\Delta z}\right),$$

where *D* is an arbitrary amplitude and *n* = 1, 2, 3,… The equivalent depths in this case are identical to those of Equation 7. Hence, as in the case of *w* = 0 at both bottom and top boundaries, the *n* = 0 solution is the trivial *L*_0_(*z*) = 0 solution.

Similarly, in the case when the top and bottom identical boundary conditions are *L*_*n*_ ′ (*z*_bot_) = 0 = *L*_*n*_ ′ (*z*_top_) there are only internal modes given by

(A3) $$\begin{array}{cc} L_{n}\left(z\right) & ={De}^{\frac{1}{2H_{0}}z}\left(\cos\left(\frac{\pi n\left(z-z_{\mathrm{bot}}\right)}{\Delta z}\right)\right.\\ & \left.\;-\frac{\Delta z}{2H_{0}\pi n}\sin\left(\frac{\pi n\left(z-z_{\mathrm{bot}}\right)}{\Delta z}\right)\right) \end{array}$$

where *D* is an arbitrary amplitude and *n* = 1, 2, 3, … with the same equivalent depths as of Equation 7 (and the *n* = 0 solution is the trivial *L*_0_(*z*) = 0 solution).

Thus, in all three combinations of identical top and bottom boundary conditions there are no *n* = 0 internal (baroclinic) modes.

## 2 Mixed boundary conditions

The expressions for *G*(*x*) of Equation 9 for the different combinations of mixed boundary conditions for *h*_*n*_ < 4*κH*_0_ are:

$$G\left(x\right)=\frac{2H_{0}}{\Delta z}\cdot \left\{\begin{array}{ccc} \mp \;1 & \begin{array}{l} \text{when}\;L_{n}=0\\ \text{and}\;L_{n}^{\prime }=0 \end{array} & \\ \mp \frac{2\kappa }{\left(1-2\kappa +{\left(\frac{2H_{0}}{\Delta z}x\right)}^{2}\right)} & \begin{array}{l} \text{when}\;L_{n}=0\\ \text{and}\;w=0 \end{array} & \begin{array}{l} G\left(x\right)\overset{x\to 0}{\to }\mp \frac{4}{3}\frac{2H_{0}}{\Delta z}\\ G\left(x\right)\overset{x\to \infty }{\to }\;0 \end{array}\\ \pm \frac{\left(1-4\kappa +{\left(\frac{2H_{0}}{\Delta z}x\right)}^{2}\right)}{\left(1-2\kappa +\left(1+2\kappa \right){\left(\frac{2H_{0}}{\Delta z}x\right)}^{2}\right)} & \begin{array}{l} \text{when}\;L_{n}^{\prime }=0\\ \text{and}\;w=0 \end{array} & \begin{array}{l} G\left(x\right)\overset{x\to 0}{\to }\mp \frac{1}{3}\frac{2H_{0}}{\Delta z}\\ G\left(x\right)\overset{x\to \infty }{\to }\pm \frac{7}{11}\frac{2H_{0}}{\Delta z} \end{array} \end{array}\right.\;\;$$

Remarks:

1. The sign of *G*(*x*) in these expressions is determined by the point where each of the boundary conditions is applied, *z*_bot_ and *z*_top_: the upper sign belongs to the case when the upper variable vanishes at *z*_bot_ and the lower variable vanishes at *z*_top_. The lower sign applies in the opposite case.
2. The limits of *G*(*x*) at *x* → 0 or *x* → ∞ (last column) are calculated for $\kappa =\frac{2}{7}$.

For *h*_*n*_ > 4*κH*_0_, *G*(*x*) of Equation 10 has the same form as for *h*_*n*_ < 4*κH*_0_ except for the change of sign of all *x* ^2^ terms in the last two expressions of *G*(*x*) given above.

The results for all combinations of boundary conditions and temperature profiles are shown in Figures 6, 7 and A3 for the troposphere and are expansions of Figures 1, 2 and 3. For the combined troposphere‐stratosphere layer, Figure A4 is the counterpart of Figure 4.

## Appendix

For the stratosphere, the condition of *w* = 0 at the bottom is not realistic. Indeed, there is no well‐justified boundary condition to apply in such a situation, and some studies elect to set geopotential height equal to a constant value (Holton and Mass, 1976; Gray *et al*., 2003) while others set the upward propagating Eliassen–Palm flux to a constant value (Sjoberg and Birner, 2014). Here, we chose the boundary condition according to mathematical considerations related to the form of a general Sturm–Liouville problem to be the vanishing of either *L* or *L*', that is, Dirichlet or Neumann condition at the lower boundary (see e.g. Arfken (1985)). Note that a constant geopotential height can be related to *L* and *L*' (see eq. 35 of Lindzen and Chapman (1969)). According to the Sturm–Liouville theorem, such conditions can shed a light on the characteristics of the eigenfunctions and eigenvalues of the vertical equation, even if the equation is not fully solvable (Arfken, 1985), in contrast to cases of boundary conditions that consist of a linear combination of Dirichlet and Neumann conditions, for example, *w* = 0.

The stratosphere can be treated separately as an isolated layer since there are significant differences between its temperature profile and the temperature profiles in adjacent layers, and there is a long history of modelling studies focusing on stratospheric dynamics with a lower boundary near the tropopause (Holton and Mass, 1976; Gray *et al*., 2003; Sjoberg and Birner, 2014).

### 1 Results —stratosphere

We solve Equation 1 numerically for four temperature profiles: (a) a realistic profile based on the US Standard Atmosphere, 1976; (b) a parabolic profile: $T\left(z\right)=220+\frac{270-220}{{\left(\Delta z\right)}^{2}}{\left(z-z_{\mathrm{bot}}\right)}^{2}$; (c) a linear profile: $T\left(z\right)=220+\frac{270-220}{\Delta z}\left(z-z_{\mathrm{bot}}\right)$; and (d) a constant profile with (*z*) = *T*_0_ =236 K – the (height‐)weighted mean of the realistic profile between 220 and 270 K.

The relevant boundary conditions in the stratosphere are those with either *L* = 0 or *L*' = 0 at the bottom and top. The condition of *w* = 0 at the layer's bottom is not realistic since the tropopause is not a material surface though the stratosphere can be approximated as a dynamically isolated layer. Also, the condition of *w* = 0 at the layer's top is not realistic because the stratopause is neither a material surface nor is it located at infinity. However, we consider also the conditions of *w* = 0 at the bottom or at the top though it is not realistic since it has an exact analytic (or semi‐analytic) solution (for constant temperature), and other cases can be compared to this analytic solution. Therefore, as in the troposphere, we extend our analysis to all nine combinations of boundary conditions in order to better understand the classification of the different modes.

For constant temperature profile, and as in the troposphere, the agreement is excellent between the analytic (or semi‐analytic) and numerical solutions. In contrast to the troposphere, no baroclinic *n* = 0 mode exist (i.e. no intersections of the RHS of Equation 9 with the first branch of the tangent function; results not shown) since the layer thickness is too large (and the mean temperature not low enough) so *G*(0) in Equation 9 is smaller than 1 – the slope of tan(*x*)|_*x* = 0_. Three combinations of mixed boundary conditions yield positive RHS of Equation 10 that can intersect the positive half of the tanh(*x*) function, but only for two of them (with *w*(*z*_bot_) = 0) does the intersection occur at *x* values associated with *h*_*n*_ values that are not much larger than the layer's physical thickness. These modes would be recognized as barotropic modes if the *w*(*z*_bot_) = 0 boundary condition were acceptable; it resembles the analytic barotropic mode both in its associated equivalent depth and in the monotonic increase of its eigenfunction in the lower 10 km which is almost identical to the analytic eigenfunction. The third intersection (associated with the *L*'(*z*_bot_) = 0 and *L*(*z*_top_) = 0 boundary condition combination) yields *h*_*n*_ of O(100 km) which is much larger than the layer's physical thickness (results not shown).

For the four different temperature profiles, Figure B1 compares the equivalent depths for different combinations of boundary conditions. The left panel shows the equivalent depths of the barotropic mode (which is not relevant to the stratosphere since this mode only exists when a solid boundary bounds the layer from below) and the right panel shows the *n* = 1 baroclinic modes. The values of barotropic equivalent depths on the left panel (9,300 ± 500 m) are very similar to each other and of the same order as the analytic value of 9,666 m calculated (analytically and numerically) for constant temperature (the slight difference between this analytic value of 9,666 m and the tropospheric value of 10,403 m originates from the different mean temperature accounted for). The equivalent depth of the *n* = 1 baroclinic mode ranges from 2,300 m to 5,300 m depending on the precise boundary conditions implemented, and this range encompasses the analytic solution of 3,323 m of the three combinations of identical boundary conditions for which an analytic solution is derived for constant temperature.

Two key results are evident in Figure B1:

1. The details of the temperature structure are not important when calculating the equivalent depth: for a given boundary condition combination the values of *h*_1_ differ by less than 10% among the four temperature profiles.
2. The equivalent depths are sensitive to the boundary conditions used and can differ by up to a factor of two for a given temperature profile. In all 36 cases the equivalent depth ranges between ∼2,300 and 5,200 m (*h*_1_ = 3,700 m ± 1,500 m), i.e. an overall variation of less than 50%. These findings imply that the equivalent depth of the stratosphere is unique and cannot be estimated simply by extrapolating the values in the troposphere. In contrast, the eigenfunctions in the stratosphere are very similar to those of the troposphere shown in Figure A3 (up to stretching) so the results are not shown.

The results also imply that the dynamics of a barotropic sudden warming must include a tropospheric component as well, as only the combined troposphere‐stratosphere configuration supports a barotropic mode. Indeed, in the for example January 2009 warming event, a wave‐2 structure was present all the way from the troposphere to the stratosphere (Harada *et al*., 2010). In light of these findings, the appropriate way to interpret the shallow‐water models for barotropic sudden warmings (Esler and Scott, 2005; Matthewman and Esler, 2011; Liu and Scott, 2015; Scott, 2016) is that these models represent a coupled troposphere and stratosphere configuration (consistent with Matthewman *et al*., 2009). It is also conceivable that nonlinear dynamics within the stratosphere are important for a barotropic sudden warming, and our analysis in this article concerns the linearized primitive equations where nonlinear effects are excluded.
